# Safety and efficacy of pulse-induced contour cardiac output monitoring in elderly patients with coronary artery disease and severe heart failure at coronary care units

**DOI:** 10.3389/fcvm.2022.910898

**Published:** 2022-10-19

**Authors:** Qi Li-ping, Liu Hong-wei, Hong Chang-ming, Bai Yong-yi, Li Ang

**Affiliations:** Department of Cardiology, National Center for Clinical Medicine of Geriatric Diseases, The Second Clinical Center, Chinese PLA General Hospital, Beijing, China

**Keywords:** hemodynamic monitoring, geriatric patients, pulse-induced contour cardiac output, heart failure, coronary artery disease

## Abstract

**Background:**

The optimal treatment for elderly patients with severe heart failure depends on the accurate assessment of their hemodynamic status. Due to its less invasive nature, the safety and efficacy of invasive pulse-induced contour cardiac output (PiCCO)-based hemodynamic monitoring remains uncertain.

**Methods:**

This was a prospective observational study. Between January 2016 and July 2020, 190 elderly patients with severe heart failure were consecutively enrolled. The PiCCO group (89 patients) and non-invasive hemodynamic monitoring group (101 patients) were observed. Hospital stays results were evaluated.

**Results:**

No significant difference in clinical data (*P* > 0.05) or the incidence of 1-month mortality (16.0 vs. 35.0%, *P* = 0.141) were observed between groups. The coronary care unit (CCU) stay was shorter in the PiCCO group than in the non-invasive group (40.0 vs. 43.0%, *P* = 0.049). Indicators such as low Extravascular Lung Water Index (EVLWI), high Body Mass Index (BMI), low Pulmonary Artery Pressure (PAP), and high Left Ventricular Ejection Time (LVET), were associated with favorable clinical results.

**Conclusion:**

Early invasive PiCCO monitoring is safe in critically ill elderly patients with severe heart failure. The hospital stay was reduced using PiCCO monitoring. These encouraging PiCCO results favor its use in elderly patients with severe heart failure at CCUs.

## Introduction

Water-sodium retention is the main cause of hospital admission for patients with heart failure, and volume management is the standard of care for heart failure (1–3). Hemodynamic monitoring is of great significance for patients with severe heart failure in order to provide volume management and organ perfusion therapy. It also plays a key role in individualized treatment.

However, common indicators, such as central venous pressure (CVP) and pulmonary capillary wedge pressure (PCWP), cannot accurately reflect volume status because they are affected by myocardial compliance, ventilator, respiratory movement, and thoracic and abdominal pressure (4, 5). While indicators measured using a pulmonary artery catheter are the gold standard, this method’s application is limited due to safety concerns and cost considerations.

Advances in technology have resulted in new procedures, such as pulse-induced contour cardiac output (PiCCO), which can offer precise monitoring of cardiovascular functions and hemodynamic parameters (6–8). Compared to the traditional invasive right heart catheterization, PiCCO has the advantages of simple operation, safety, and long indwelling time (4, 5). In addition, thoracic electrical bioimpedance (TEB) and transthoracic echocardiography are two commonly used methods for non-invasive hemodynamic monitoring (9, 10).

At present, PiCCO and non-invasive TEB-based hemodynamic monitoring are used to monitor and guide the treatment of patients with severe heart failure and shock. Which of the two methods is better has not been studied in elderly patients with severe heart failure. Data on elderly patients with heart failure is lacking, so the effect of early hemodynamic monitoring on the results in these patients and safety of invasive PiCCO monitoring for clinical treatment guidance are unknown.

## Material and methods

The study protocol was approved by the Ethics Review Board of Chinese PLA general hospital (Beijing, China).

### Ethics statement

These authors are accountable for all aspects of the work in ensuring that questions related to the accuracy or integrity of any part of the work are appropriately investigated and resolved. The trial was conducted in accordance with the Declaration of Helsinki (as revised in 2013). The study was approved by the Ethics Review Board of Chinese PLA general hospital (Beijing, China) (NO. 16BJZ22) and informed consent was taken from all individual participants.

### Subjects

Elderly patients with coronary heart disease and severe heart failure were consecutively enrolled in the present study. PiCCO and non-invasive hemodynamic indicators were measured and relationships among these indicators and adverse events were prospectively observed in order to evaluate the safety of invasive PiCCO monitoring and correlation between monitoring index and hospital results in elderly patients with coronary artery disease and severe heart failure. Each patient or his/her family signed the informed consent before the study.

A total of 190 elderly patients with severe heart failure from CCU admitted to the Second Clinical Center of Chinese PLA general hospital between January 2016 and December 2020 were consecutively enrolled in the study. The PiCCO group included 89 patients aged 68–96 years, with a mean age of 82.30 ± 10.70 years. The non-invasive group included 101 patients aged 67–95 years, with a mean age of 81.10 ± 8.60 years.

### Inclusion and exclusion criteria

Inclusion criteria were as follows: known history of coronary heart disease; orthopnea (inability to lie down); wet rales in the lungs; edema in the lower extremities; echocardiography showing left ventricular end-diastolic diameter of >50 mm and left ventricular ejection fraction (LVEF) of <50%, or chest X-ray showing pulmonary congestion or edema; and type I respiratory failure (partial pressure of oxygen of <50 mm Hg even after oxygen therapy, requiring tracheal intubation and mechanical ventilation after conventional treatments, such as cardiotonic therapy, diuretics, and vasoactive drugs). Exclusion criteria were as follows: heart failure with uncontrolled severe infection, and pulmonary diseases.

### Study design

This was a prospective observational study with two study groups: the PiCCO group and the non-invasive group.

### Groups and indicators

In the PiCCO group, a central venous catheter was inserted into the subclavian vein, and an artery thermistor catheter was inserted into the femoral artery. The PiCCO2 monitor (Pulsion Medical Systems, Munich, Germany) was then connected. After a bolus injection of 15 mL of ice-cold saline via the central venous line, the indicators were measured three times, and the mean value was recorded for further analysis. Specifically, CI (cardiac index), GEDVI (global end-diastolic volume index), EVLWI, and systemic vascular resistance index were measured to guide volume management.

In the non-invasive group, the BioZ digital non-invasive hemodynamic monitor (Cardio Dynamics, USA) was used. The patient’s height, weight, age, and sex were entered when prompted on the monitor screen. Monitoring began once the cardiac function curve was steady. Monitored indicators included CI, systemic vascular resistance (SVR), acceleration index (ACI; myocardial contractility index), thoracic fluid content (TFC), systolic time ratio (STR), LVET, and pre-ejection period (PEP). Each indicator was measured three times, and the mean value was recorded for further analysis.

A central venous line was inserted via the subclavian vein to monitor CVP in both groups. In addition, echocardiography was performed (patient position: hip angle of 30^°^, half-seating position) to measure LVEF, LVET, and PAP. All echocardiography exams were performed by the same experienced sonographer.

A blood sample was collected from both groups on the same day as hemodynamic monitoring to measure N-terminal pro-B-type natriuretic peptide (NT-proBNP), myocardial marker cardiac troponin I/T (cTnI/T), serum creatinine (SCr), and serum albumin (Alb) at the clinical laboratory of Chinese PLA general hospital.

### Treatments

For the PiCCO group, the normal reference ranges were 680–800 mL/m^2^ for GEDVI and 3.0–7.0 mL/kg for EVLWI. PiCCO monitoring was discontinued after EVLWI had been stable for at least 2 days.

For the non-invasive group, the normal reference ranges were 4–8 L/min for CO, 2.5–4.2 L/min/m^2^ for CI, 770–1,500 dynes/s/cm^–5^ for SVR, 70–150 s^2^ (male) and 90–170 s^2^ (female) for ACI, 30–45 Ω^–1^ (male) and 21–37 Ω^–1^ (female) for TFC, 0.3–0.5 for STR, 50–120 ms for PEP, and 250–350 ms for LVET. CVP was kept at 8–10 mmHg, and the volume was managed based on signs of pulmonary edema on a chest X-ray and NT-proBNP level.

Hypoproteinemia was corrected in both groups to keep albumin >30 g/L. Loop diuretics (i.v. intravenous injection), aldosterone receptor antagonists (p.o. *per os*), and recombinant human BNP (i.v. intravenous injection), as well as vasoactive drugs and positive inotropic drugs, were used to manage volume. Two groups were treated with full course according to the course of recombinant human BNP, and loop diuretics as needed. In addition, measures were taken to prevent and treat infection.

Based on the PiCCO monitoring, decision tree, and clinical symptoms, patients with low CI or CO received cardiotonic therapy until CI or CO improved. Patients with low GEDVI received rehydration therapy (fluid replenishment). Patients with high EVLWI received loop diuretics (i.v.), aldosterone receptor antagonists (p.o.), and recombinant human BNP (i.v.) to rapidly reduce the circulating volume and improve cardiac function.

Clinical and hospital medical records were reviewed as part of in-hospital results to collect information about serious cardiac events, including unstable angina, myocardial infarction, revascularization, heart failure, and cardiac death. A 3-month hospital stay was considered a minor adverse event.

### Statistical analysis

SPSS v22.0 (IBM SPSS Statistics 22) was used for data analysis. Normally distributed measurement data were expressed as mean ± standard deviation and analyzed using a Student’s *t*-test. Non-normal distribution data were expressed according to the quartile, and the non-parametric test method is applied. Analysis of variance was performed to compare time points. Cox regression analysis was used to describe outcome data. *P* < 0.05 was considered statistically significant.

## Results

### In-hospital mortality and morbidity

No significant difference in age, sex, body mass index (BMI), left cardiac indices (per echocardiography), SCr (kidney function), serum albumin, NT-proBNP (cardiac function), or CVP (myocardial injury, volume status) was observed between the PiCCO and non-invasive groups (*P* > 0.05; Table 1). The incidence of serious 1-month cardiac events (myocardial infarction, revascularization, heart failure, and cardiac death) did not differ between groups (44 vs. 40%, *P* = 0.787; Table 2). The 1-month death rate wasn’t difference between the two groups (16.0 vs. 35.0%, *P* = 0.141) in elderly patients with severe heart failure (Table 2). The difference in the 1-month death rate was not statistically significant, which may be related to the small sample size. However, the CCU stay was shorter in the PiCCO group than in the non-invasive group (40.0 vs. 43.0%, *P* = 0.049; Table 2).

**TABLE 1:** Clinical data for PiCCO and non-invasive groups.

Variables	PiCCO group (*n* = 89)	Non-invasive group (*n* = 101)	*P*
Age (year)	82.30 ± 10.70	81.10 ± 8.60	0.679
Sex (%)	10 (12%)	10 (10%)	0.701
BMI (kg/m^2^)	21.46 ± 3.60	22.94 ± 3.80	0.190
NT-proBNP (pg/ml)	3276.51 (1591.50, 11372.0)	2357.15 (1328.07, 7014.29)	0.371
cTnI (μg/L)	0.62 (0.31, 1.88)	1.20 (0.60, 3.61)	0.342
CTnT (ng/ml)	0.20 (0.08, 0.77)	0.27 (0.12, 0.97)	0.738
SCr (μmol/L)	142.64 ± 86.83	102.37 ± 53.64	0.080
Alb (g/L)	29.45 ± 2.21	28.45 ± 4.02	0.460
Ultrasound LVEF (%)	40.60 ± 9.00	45.70 ± 7.20	0.534
Ultrasound LVET (ms)	274.00 ± 18.19	277.42 ± 24.30	0.596
Ultrasound AP (mmHg)	38.72 ± 10.61	37.63 ± 12.18	0.753
CVP (cmH_2_O)	13.96 ± 6.78	12.74 ± 5.71	0.430

PiCCO, pulse-induced contour cardiac output; BMI, body mass index; NT-proBNP, N-terminal pro b-type natriuretic peptide; cTNI/T, cardiac troponin I/T; SCr, serum creatinine; Alb, albumin; LVEF, left ventricular ejection fraction; LVET, left ventricular ejection time; PAP, pulmonary artery pressure; CVP, central venous pressure.

**TABLE 2:** Follow-up data for the two groups.

Group	Hospital stay	Cardiac events	1-month mortality
PiCCO group	40%	44%	16%
Non-invasive group	43%	40%	35%
*P*	0.049	0.787	0.141

PiCCO, pulse-induced contour cardiac output.

In the multivariate Cox regression analysis of 1-month adverse effects in the two groups, PiCCO monitoring was an independent predictor of survival in elderly patients with coronary heart disease and severe heart failure [hazard ratio (HR) = 0.54, 95% confidence interval (CI) 0.087–1.45, *P* = 0.187) after controlling for age, sex, and weight.

### Correlation between 1-month mortality endpoint and hemodynamic, invasive, and non-invasive pulse-induced contour cardiac output indicators

Stepwise regression analysis of all hemodynamic indicators showed that BMI and ultrasound PAP (uPAP) were correlated with the 1-month mortality endpoint. BMI and PAP were independent risk factors for minor adverse events. BMI was negatively correlated with 1-month mortality (HR = 0.808, 95%CI 0.683–0.956, *P* = 0.013; Table 3), and uPAP was positively correlated with 1-month mortality (HR = 1.052, 95%CI 1.009–1.096, *P* = 0.017; Table 3).

**TABLE 3:** Correlations between indicators and 1-month mortality endpoint (multivariate analysis).

	HR (95% CI)	*P*-value
BMI	0.808 (0.683–0.956)	0.013
uPAP	1.052 (1.009–1.096)	0.017
CTnI	0.963 (0.735–1.260)	0.782
CTnT	1.087 (0.189–6.250)	0.926
NT-proBNP	1.000 (1.000–1.000)	0.771
SCr	1.001 (0.824–0.992)	0.824
LVET	0.968 (0.937–0.999)	0.045
PAP	1.088 (1.028–1.152)	0.004
EVLWI	1.390 (1.007–1.919)	0.045

HR, hazard ratio; CI, confidence interval; BMI, body mass index; cTnI/T, cardiac troponin I/T; NT-proBNP, N-terminal pro b-type natriuretic peptide; SCr, serum creatinine; LVET, left ventricular ejection time; uPAP, ultrasound pulmonary artery pressure; EVLWI, extravascular lung water index.

With regard to invasive PiCCO indicators, EVLWI (HR = 1.390, 95%CI 1.007–1.071, *P* = 0.045) was an independent risk factor for 1-month mortality after controlling for age, sex, and weight using multivariate Cox regression analysis (Table 3). For non-invasive indicators, PAP (HR = 1.088, 95%CI 1.028–1.152, *P* = 0.004) and LVET (HR = 0.968, 95%CI 0.937–0.999, *P* = 0.045) were independent risk factors for 1-month mortality (Table 3).

## Discussion

This clinical study is the first real-world prospective observational study to investigate the safety and efficacy of early PiCCO monitoring in elderly patients with coronary heart disease and severe heart failure. The results showed that invasive PiCCO monitoring reduced the risk of hospital/CCU stay without increasing the incidence of 1-month mortality, which is consistent with other reports (11, 12). PiCCO indicators reflect cardiac function in order to precisely and accurately diagnose, treat, and prevent. PiCCO monitoring is superior to conventional CVP monitoring in helping to improve the symptoms and prognosis of patients with shock-induced hemodynamic instability (13).

The value of PiCCO monitoring for improving patient outcomes is still under debate. PiCCO monitoring can be used to guide volume support, rehydration, and cardiotonic therapy, thereby aiding individualized management to protect the heart, kidneys, and lungs and achieve timely and effective reversal of the pathophysiological changes occurring during heart failure (14–16). Early intervention and targeted treatment were guided, thus improving patient conditions. Some studies have found that among patients undergoing abdominal emergency surgery, the incidence of surgical complications was higher and the prognosis was worse in the PiCCO monitoring group than in the conventional hemodynamic monitoring group (11), and that PiCCO monitoring did not reduce the incidence of kidney failure in patients undergoing abdominal surgery (13, 16). Other studies have shown that PiCCO monitoring helped to reduce postoperative complications and hospital stay duration with no apparent effect on mortality or patient outcomes. This discrepancy may be due to intervention delays associated with a high treatment threshold or mismatches between the threshold and patient condition. Taken together, most evidence shows that PiCCO monitoring improves prognosis, although it appears to have no effect on mortality and morbidity (11). This was also demonstrated by the present results, where the CCU stay was shorter in the PiCCO group than in the non-invasive group. PiCCO group has potential economic benefits.

Hemodynamic indicators are correlated with prognosis. EVLWI was positively correlated with 1-month mortality in the present study. PiCCO monitoring can detect a small increase in EVLWI (10–15%), while chest X-ray is negative until EVLWI has increased by 100–300%. Because it is far more sensitive than chest X-ray in evaluating pulmonary edema, EVLWI is a better indicator for guiding early use of diuretics to actively reduce fluid load in the body, thereby improving the symptoms and prognosis in heart failure patients (9) EVLWI is correlated with prognosis in shock patients and positively correlated with mortality (17). EVLWI may also be used to guide diuretic therapy to improve the survival and prognosis in patients with heart failure or shock (18). EVLWI is an independent risk factor and can be used as an auxiliary indicator for prognosis in elderly patients with severe heart failure.

The present study also demonstrated that other hemodynamic indicators were correlated with prognosis. A low BMI is an independent risk factor for 1-month mortality in patients with post-infarction heart failure. It also indicates poor life quality, which has been illustrated by prior studies (19). PAP is also an independent risk factor for 1-month mortality, although some studies have shown that PAP is uncorrelated with adverse prognosis (20). However, multifactor regression analysis showed that PAP was correlated with adverse prognosis in the present and other studies (21). In addition, LVET has been shown to be an independent predictor of heart failure and death (22), which was also noted by the present study. In summary, low ELWI, high BMI, low PAP, and high LVET are associated with favorable results.

All of the patients participated in the hospital investigation and provided complete data, thereby providing strong evidence for the present conclusion. The data showed that PiCCO reduced the 1-month hospital mortality and hospital stay. The reasons for this decrease were based on the PiCCO monitoring, decision tree, and clinical symptoms. Patients with low CI received cardiotonic therapy, those with low GEDVI received rehydration therapy (fluid replenishment), and patients with high EVLWI received loop diuretics (i.v.) to rapidly reduce the circulating volume and improve cardiac function.

This study had some limitations. The threshold (per decision tree) had to be combined with clinical symptoms in order to enable individualized treatment. In this prospective observational study, the two groups of patients were comparable. However, it was a small, single-center study with a short follow-up time. Studies with a larger sample size and longer duration are needed to validate the present results.

In summary, this prospective observational study demonstrated that PiCCO monitoring is safe and can be effectively used for early monitoring of elderly patients with severe heart failure.

## Data availability statement

The original contributions presented in this study are included in the article/supplementary material, further inquiries can be directed to the corresponding author/s.

## Ethics statement

The studies involving human participants were reviewed and approved by the Ethics Review Board of Chinese PLA general hospital (Beijing, China). Study protocol number: No. 16BJZ22. The patients/participants provided their written informed consent to participate in this study.

## Author contributions

QL-P contributed to the study design, data acquisition, and analysis, and drafted the manuscript. LH-W contributed to the study design, data acquisition, and revision of the manuscript. HC-M and LA were involved in data acquisition. BY-Y contributed to the data-analysis. All authors contributed to the article and approved the submitted version.

## References

[1] Braunwald E. The war against heart failure: the lancet lecture. *Lancet.* (2015) 385:812–24. 10.1016/S0140-6736(14)61889-4 25467564

[2] Yancy CW Jessup M Bozkurt B Butler J Casey DE Jr Drazner MH 2013 ACCF/AHA guideline for the management of heart failure: a report of the American College of Cardiology Foundation/American Heart Association task force on practice guidelines. *J Am Coll Cardiol.* (2013) 62:e147–239. 23747642 10.1016/j.jacc.2013.05.019

[3] Koniari K Parissis J Paraskevaidis I Anastasiou-Nana M. Treating volume overload in acutely decompensated heart failure: established and novel therapeutic approaches. *Eur Heart J Acute Cardiovasc Care.* (2012) 1:256–68. 10.1177/2048872612457044 24062916 PMC3760543

[4] Su WL Shui HA Lan CC Yang MC Hsieh CA Jang SJ Cardiovascular parameters associated with troponin I as indicators for 14-day mortality in patients with septic shock. *Am J Med Sci.* (2018) 356:244–53. 10.1016/j.amjms.2018.05.008 30286819

[5] Akselrod BA Tolstova LA Pshenichniy TA Fedulova SV. Pulse wave transit time-one more attempt of non-invasive cardiac output measurement. *Anesteziol Reanimatol.* (2017) 61:178–82. 29465201

[6] Wernly B Lichtenauer M Franz M Fritzenwanger M Kabisch B Figulla HR Pulse contour cardiac output monitoring in acute heart failure patients. *Wien Klin Wochenschr.* (2016) 128:864–9. 10.1007/s00508-016-1048-z 27525745 PMC5161758

[7] Burn and Trauma Branch of Chinese Geriatrics Society Zhang WY Wang W. [National experts consensus on the application of pulse contour cardiac output monitoring technique in severe burn treatment (2018 version)]. *Zhonghua Shao Shang Za Zhi.* (2018) 34:776–81. 30481917 10.3760/cma.j.issn.1009-2587.2018.11.011

[8] Kushimoto S Endo T Yamanouchi S Sakamoto T Ishikura H Kitazawa Y Relationship between extravascular lung water and severity categories of acute respiratory distress syndrome by the Berlin definition. *Crit Care.* (2013) 17:R132. 10.1186/cc12811 23844662 PMC4056600

[9] Wernly B Haumann S Masyuk M Muessig J Lichtenauer M Laura Bäz L Extravascular lung water index and Halperin score to predict outcome in critically ill patients. *Wien Klin Wochenschr.* (2018) 130:505–10. 10.1007/s00508-018-1370-8 30094662

[10] Bour J Kellett J. Impedance cardiography:a rapid and cost-effective screening tool for cardiac disease. *Eur J Intern Med.* (2008) 19:399–405. 10.1016/j.ejim.2007.07.007 18848172

[11] Zhang YB Zhang ZZ Li JX Wang YH Zhang WL Tian XL Application of pulse index continuous cardiac output system in elderly patients with acute myocardial infarction complicated by cardiogenic shock: a prospective randomized study. *World J Clin Cases.* (2019) 7:1291–301. 10.12998/wjcc.v7.i11.1291 31236393 PMC6580342

[12] Zhang Y-B Zhang Z-Z Li J-X Wang Y-H Zhang W-L Tian X-L Application of pulse index continuous cardiacoutput system in elderly patients with acute myocardial infarction complicated by cardiogenic shock: A prospective randomized study. *World J Clin Cases* (2019) 7:1291–301. 10.12998/wjcc.v7.i11.1291 31236393 PMC6580342

[13] Pavlovic G Diaper J Ellenberger C Frei A Bendjelid K Bonhomme F Impact of early haemodynamic goal-directed therapy in patients undergoing emergency surgery: an open prospective, randomised trial. *J Clin Monit Comput.* (2016) 30:87–99. 25851818 10.1007/s10877-015-9691-x

[14] Phan TD D’Souza B Rattray MJ Johnston MJ Cowie BS. A randomised controlled trial of fluid restriction compared to oesophageal doppler-guided goal-directed fluid therapy in elective major colorectal surgery within an enhanced recovery after surgery program. *Anaesth Intensive Care.* (2014) 42:752–60. 10.1177/0310057X1404200611 25342408

[15] Scheeren TW Wiesenack C Gerlach H Marx G. Goal-Directed intraoperative fluid therapy guided by stroke volume and its variation in high-risk surgical patients: a prospective randomized multicentre study. *J Clin Monit Comput.* (2013) 27:225–33. 10.1007/s10877-013-9461-6 23558909

[16] Schmid S Kapfer B Heim M Bogdanski R Anetsberger A Blobner M Algorithm-guided goal-directed haemodynamic therapy does not improve renal function after major abdominal surgery compared to good standard clinical care: a prospective randomised trial. *Crit Care.* (2016) 20:50. 10.1186/s13054-016-1237-1 26951105 PMC4782303

[17] Wang H Cui N Su L Long Y Wang X Zhou X Prognostic value of extravascular lung water and its potential role in guiding fluid therapy in septic shock after initial resuscitation. *J Crit Care.* (2016) 33:106–13. 10.1016/j.jcrc.2016.02.011 27021852

[18] Pan P Su LX Zhou X Long Y Liu DW Wang XT. Critical Hemodynamic Therapy-oriented resuscitation helping reduce lung water production and improve survival. *Chin Med J.* (2019) 132:1139–46. 10.1097/CM9.0000000000000205 30882456 PMC6511433

[19] Stienen S Ferreira JP Girerd N Duarte K Lamiral Z McMurray JJV. Mean BMI, visit-to-visit BMI variability and BMI changes during follow-up in patients with acute myocardial infarction with systolic dysfunction and/or heart failure: insights from the high-risk myocardial infarction initiative. *Clin Res Cardiol.* (2019) 108:1215–25. 10.1007/s00392-019-01453-7 30953180

[20] Nagaoka M Goda A Takeuchi K Kikuchi H Finger M Inami T Nocturnal hypoxemia, but not sleep apnea, is associated with a poor prognosis in patients with pulmonary arterial hypertension. *Circ J.* (2018) 82:3076–81. 30333436 10.1253/circj.CJ-18-0636

[21] Tao K Ishikawa Y Inage A Ueda T Kishiki K Yoshikawa T Medium-remote term results of the atrioventricular valve replacement with mechanical valve for functional single ventricles. *Kyobu Geka.* (2018) 71:650–7. 30185737

[22] Biering-Sørensen T Querejeta Roca G Hegde SM Shah AM Claggett B Mosley TH Jr Left ventricular ejection time is an independent predictor of incident heart failure in a Community-Based cohort. *Eur J Heart Fail.* (2018) 20:1106–14. 10.1002/ejhf.928 28872225 PMC6685547

